# The Suppression of Quackery

**Published:** 1897-06-05

**Authors:** 


### The Hospital

A Journal of

The Medical Sciences and Hospital Administration.

Vol. XXII.—No. 558.] [June 5, 1897.

### The Suppression of Quackery.

The General Medical Council, during the session which has just been brought to a close, has been engaged in a laudable attempt to obtain from Parliament powers for the suppression of quackery—powers which the Medical Act of 1858 was supposed to be intended to provide, but which, as a matter of fact, it failed to provide effectually. It enacted by its fortieth section, that any person, not duly qualified and registered as a medical practitioner, who used any title, or pursued any course of action calculated to induce persons to believe that he was registered under the Act, might be prosecuted summarily, and sentenced, on conviction before a magistrate, to a penalty not exceeding twenty pounds. It also enacted, by its forty-second section, that any sum or sums of money arising from conviction and recovery of penalties as aforesaid, should be paid to the Treasurer of the General Medical Council. If these provisions had been realities, they would have furnished a most effective weapon against fraudulent pretenders to medical status and to medical knowledge; but, as matters turned out, they were wholly illusory, and broke in the hands of whoever attempted to employ them. The first-named section, by which the offence is defined, is qualified or diluted by the words "wilfully and falsely," and many years elapsed before the average magistrate could be brought to infer the wilfulness of a course which was obviously false, and which was systematically pursued. When at last, in some very flagrant case, a penalty was inflicted, the money, instead of being paid to the Council, as directed by the Act, was claimed by the Receiver of the Metropolitan Police in every instance in which the offence had been committed within the metropolitan district, the provision of the Medical Act being of no avail against that of the earlier Police Act, in the absence of special words in the former by which the operation of the latter would be suspended. Nor was the Receiver of the Metropolitan Police alone; for control over fines was found to be claimed by the police under local Acts in various provincial towns and districts. The general result has been that in all the successful London prosecutions, and in some of those which have been carried to conviction in provincial towns, the accused persons have indeed been fined, but the prosecuting individual or body has been called upon to pay the whole of the expenses, which have in many instances been so considerable as to render it impossible to pursue the numerous offenders in any effectual or systematic manner.

It is said of an eminent engineer, who was also a dilettante in sanitary matters, that he once applied himself to a diligent study of the history of a cholera epidemic which visited parts of southern Russia, and that he subsequently announced to the world, as a discovery, that cholera did not spread in uninhabited regions. What is true of cholera is at least equally true of the more injurious forms of quackery; and, in order to see these in their epidemic development, it is necessary to visit the great centres of population. The special Police Acts to which we have referred are, probably in every case, town Acts; and each of them is to this extent a shield of defence to the quack, who does not carry on his trade in villages or in rural districts. The Medical Defence Union has made several endeavours to enforce what it at first supposed to be law; and has not only encountered some nasty defeats by reason of the "wilfully and falsely" provision, but also, even when successful, has been too seriously out of pocket to be able to indulge in similar prosecutions otherwise than as occasional luxuries. The Medical Council, aided by the perfect familiarity of its legal advisers with the questions at issue, has shown how such prosecutions can be successfully conducted, and, while not recognising it as a duty to institute them itself, save under exceptional conditions, has always declared its readiness to give the penalties which it received in aid of the costs of the proceedings. But for the Police Acts, this assistance would be so considerable, that it would enable the Defence Union, or any similar body, to make a clean sweep of a large proportion of the quack fraternity.

At the present time, there is a Bill before the House of Commons, and under the consideration of the Committee on Law, which, if carried, will effect certain changes in the financial position and relations of the police; and the Medical Council has memorialised the Government, and has petitioned Parliament, asking for the insertion of a clause which wonld render the forty-second section of the Medical Act operative in all parts of England, and would thus compel quacks to pay for, or at least to contribute to, the expenses of their own abolition. It is very difficult indeed adequately to estimate the amount of mischief which is done by these wretches, or the sum total of misery which they cause. Their so-called treatment does not signify much, one way or the other, being seldom sufficiently active to be in any high degree injurious; although it may, of course, occasion hurtful or even fatal delay in the application of proper remedies. The real sting of the matter, the real basis of the offence, is the extortion of money under false pretences. When this is done from the rich, or from people who have had opportunities of being educated, it is not of very much consequence; for, if they are fools, they must naturally pay the penalty in some direction, and, if they were not robbed by quacks, they would fall victims to some other kind of swindle. The really important case is that of the poor. A notorious dealer in electrical instruments was exposed a few years ago, and we heard at the time, from one of the physicians to a hospital for paralysis, that numbers of the out-patients there had pawned their goods, and had deprived themselves of necessaries, in reliance upon this man's promises of cure, and in order to buy his worthless trumpery at fabulous prices. This is really the evil for which Parliament is asked to provide a remedy; and as the police, who now receive the penalties, never prosecute these rascals themselves, the Treasury cannot lose by granting the application.

